# It’s just a breast: an examination of the effects of sexualization, sexism, and breastfeeding familiarity on evaluations of public breastfeeding

**DOI:** 10.1186/s12884-022-04436-1

**Published:** 2022-02-12

**Authors:** Yuliana Zaikman, Amy E. Houlihan

**Affiliations:** Department of Psychology and Sociology, Texas A&M University-Corpus Christi, 6300 Ocean Drive, Corpus Christi, TX 78412 USA

## Abstract

**Background:**

Despite the legal right to breastfeed in public, women may be concerned about negative reactions from others, which may in turn impact their decision to breastfeed in public. The current study examined whether women breastfeeding in public (e.g., at a coffee shop) would be evaluated differently than women breastfeeding in private (e.g., at home) and explored several explanations for the possible differences: sexualization of the female breast (including the perceivers’ gender and sexual comfort level, as well as the exposure of the breast while breastfeeding), sexist attitudes, and familiarity with breastfeeding.

**Methods:**

In August 2018, 506 adult participants, residing in the United States and recruited from Amazon Mechanical Turk, were randomly assigned to view an image of a woman breastfeeding (or not) while wearing a cover (or not), in a private or public location. Participants then completed measures of their emotional responses, perceptions, and behavioral intentions toward the woman in the image as well as their sexual comfort level, hostile and benevolent sexism, and knowledge of and experience with breastfeeding.

**Results:**

People had more favorable evaluations of breastfeeding (vs. non-breastfeeding) women, especially when they had greater sexual comfort, were more knowledgeable about breastfeeding, and were parents with at least one child who was breastfed. The location (public vs. private) and the presence or absence of a cover did not differentially influence evaluations of breastfeeding and non-breastfeeding women, nor did participants’ gender or level of sexist attitudes.

**Conclusions:**

In general, people’s evaluations of breastfeeding appear to be favorable to the degree that the location of the breastfeeding is not particularly relevant to those evaluations.

**Supplementary Information:**

The online version contains supplementary material available at 10.1186/s12884-022-04436-1.

## Background

Breastfeeding is a natural act that is widely recognized as beneficial to the health of both infants and mothers [1]. Despite the legal right to breastfeed in public, many women experience, or expect to experience, discomfort, embarrassment [2, 3], and negative reactions from others [4] when they breastfeed in public. Notably, these patterns are similar across many Western countries, including the United States (U.S. [3];), Canada [5], Australia [2], and the United Kingdom (U.K. [4];). To circumvent these negative feelings and experiences, some mothers avoid breastfeeding in public (by staying home, or by breastfeeding in a restroom or car), and others choose to cover themselves and their baby while breastfeeding in public.

Only a small amount of research to date has experimentally examined evaluations of public breastfeeding. Most notably, in 2009 Acker [6] found that American participants had more favorable evaluations of breastfeeding done in private than in public. The goal of the current research was to conceptually replicate Acker’s study to determine if evaluations of breastfeeding have changed in the years since her work was published. In addition, the current study extends this work by examining additional factors that may influence evaluations of breastfeeding mothers (e.g., the use of a cover during breastfeeding, participants’ firsthand experience with breastfeeding).

### Breastfeeding in public and early cessation of breastfeeding

Given that infants need to eat every two to three hours, it is likely that mothers need to breastfeed outside the home. However, some mothers may be uncomfortable breastfeeding in public or may be concerned about negative reactions from others, which may affect how long they breastfeed. For example, in a longitudinal study of European mothers, negative maternal attitudes toward breastfeeding in public places were associated with a decreased likelihood of having breastfed in public, which was in turn associated with earlier cessation of breastfeeding [7]. Similarly, in the U.S., maternal comfort with breastfeeding in social situations was positively correlated with intention to breastfeed exclusively (versus formula feeding or mixed feeding) and with longer intended duration of exclusive breastfeeding [3].

In their examination of reasons why American mothers stop breastfeeding, Li et al. [8] found that, although not wanting to breastfeed in public was not among the most frequently cited reasons for stopping, it was deemed an important factor in the decision to stop among some of the women, particularly those who stopped breastfeeding in the first few months. Of mothers who stopped breastfeeding when their babies were between one and two months old, 18.6% cited public breastfeeding as an important reason, but this percentage dropped to 4.6% among mothers who stopped breastfeeding after nine months. Thus, addressing concerns about public breastfeeding early on (and ensuring that mothers are supported when they choose to breastfeed in public) may be helpful in encouraging women to breastfeed for the recommended six months or longer.

### Breastfeeding attitudes

Examination of the scientific literature on breastfeeding attitudes presents mixed findings. Although some studies have documented a negative bias among Americans toward breastfeeding mothers [9], others have found that breastfeeding mothers are perceived more favorably by Americans than are bottle-feeding mothers [10]. In general, attitudes toward breastfeeding tend to be favorable among various groups, including mothers [11, 12], pregnant women and their male partners [13], and non-parents [14]. Given these mixed results, we did not make an a priori hypothesis regarding the effect of breastfeeding itself (regardless of location) would have on evaluations of women (Research Question (RQ) 1a).

Research on attitudes toward *public* breastfeeding in particular has also documented mixed results. In a study examining public opinion in the U.S. toward several breastfeeding policies, Li et al. [15] found that overall support for public breastfeeding (agreement with the statement that women should have the right to breastfeed in public places) was moderate (43.1% of participants approved), but varied by age, educational level, and region (with greater approval among younger and more educated adults, as well as adults living in the Mountain and Pacific regions of the U.S). More recently, Nouer et al. [16] reported that the percentage of American adults who feel comfortable with a woman breastfeeding in their workplace (73.8%) and shopping mall or restaurant (66.5%) increased over a four-year period (2004–2008). These figures are consistent with similar studies conducted in other countries, such as Canada [17] and Australia [18]. Although acceptance of women’s rights to breastfeed in public is fairly high in Western/industrialized countries, there is still some debate or controversy regarding *how* women should breastfeed in public (e.g., whether breastfeeding women should use a cover, find a discreet location, be mindful of others’ comfort), suggesting that public breastfeeding behaviors are shaped by cultural and social norms [19–21].

It should be noted that the majority of studies on breastfeeding attitudes are survey studies that assess agreement with general statements rather than responses to particular scenarios or images. The smaller body of research that has examined reactions to breastfeeding images has found evidence of a negative bias toward public breastfeeding. For example, Magnusson and colleagues [22] asked American men to view and rate images of women breastfeeding in four locations (one private and three public). Although ratings of all images were neutral to positive, participants rated the image of the woman breastfeeding privately at home significantly more favorably than the images of women breastfeeding in public. Additionally, in studies of Canadian young adults, participants spent less time looking at images of breastfeeding than images of bottle-feeding (Study 2) and reported feeling less comfortable viewing breastfeeding images versus bottle-feeling images, especially when the breast was visible (Study 3 [23]). Another study of Canadian young women found that although an image of breastfeeding was rated more favorably than an image of bottle-feeding, attitudes toward public breastfeeding were less favorable than attitudes toward public bottle-feeding [24]. Taken together, it seems that although many people express support of breastfeeding, including women’s right to breastfeed in public places, their reactions to images of public breastfeeding suggest that they are not entirely comfortable witnessing this behavior.

Very few studies have experimentally compared reactions to breastfeeding in public vs. private locations. A notable exception is work by Acker [6], in which participants viewed pictures of mothers breastfeeding in either a public or private location. Acker found that people had more favorable evaluations of the mother breastfeeding in private and viewed her behavior as more normal and socially appropriate; similarly, they reported stronger negative feelings in response to the mother breastfeeding in public. Therefore, we predicted a two-way interaction between breastfeeding and location, such that publicly breastfeeding women would be evaluated less favorably than privately breastfeeding women; no influence of location would be observed for the non-breastfeeding women (Hypothesis (H) 1b).

### Factors influencing reactions to public breastfeeding

Acker [6] noted three possible reasons for less favorable reactions to public breastfeeding: 1) it is an effect of the (hyper)sexualization of the female breast, 2) it reflects sexist attitudes, and 3) it is a function of a lack of familiarity with breastfeeding. In the current study, we assessed people’s sexualization of the female breast from multiple factors. We examined whether covering the breast while breastfeeding in public influences evaluations of breastfeeding women, whether men and women have different evaluations, and whether one’s general sexual comfort level affects their evaluations. Moreover, when we examined familiarity with breastfeeding, we considered both general knowledge of breastfeeding as well as actual firsthand experience (i.e., whether their own children were breastfed).

#### Sexualization of the female breast

Acker [6] postulated that the sexualization of the female breast may underly negative reactions to public breastfeeding. In Western culture, the sexual role of female breasts may be more dominant (in television, movies, advertising, etc.) than their biological role. She argued that public breastfeeding may cause discomfort because it juxtaposes the maternal and sexual roles of breasts. Similarly, objectification of women may also play a role in public breastfeeding evaluations. Little research has addressed this issue among American adults, but Johnston-Robledo et al. [25] found that young American women’s self-objectification (i.e., their internalization of the objectification of their bodies) was correlated with the belief that public breastfeeding is indecent as well as with concerns that public breastfeeding would be embarrassing. Similarly, pregnant American women who scored high on self-objectification were less comfortable with public breastfeeding [26]. Furthermore, there is some evidence that a negative bias against breastfeeding mothers is similar to the bias against women whose breasts are sexualized [9]. In an attempt to separate reactions to breasts in general (which may tap into people’s sexual focus on breasts) from reactions to the actual act of breastfeeding, the current study examined reactions to breastfeeding (and non-breastfeeding) women who were depicted either wearing a breastfeeding cover (in such a way that their breast was not exposed) or not wearing a cover (in which some of the breast was exposed). We predicted a two-way interaction between breastfeeding and cover, such that the uncovered breastfeeding women would be viewed less favorably than covered breastfeeding women. No differences (or possibly less prominent differences) would be observed for the non-breastfeeding women (H1c). Finally, it is possible that there might be some mitigation of reactions to public breastfeeding if the woman is covered, thus we predicted a three-way interaction between breastfeeding, location, and cover. Specifically, we predicted that the scenarios depicting uncovered publicly breastfeeding women would be evaluated the least favorably of all the scenarios (H1d).

#### Gender

Findings on gender differences in attitudes toward public breastfeeding are mixed. Nouer and colleagues [16] found that American women were more likely than men to report feeling comfortable with mothers breastfeeding in the workplace, but no gender difference was found when asked about breastfeeding in a shopping mall. In the U.K., women reported greater support for public breastfeeding than men did [27]. However, other studies from Australia and Canada have demonstrated that women are *less* likely than men to say that breastfeeding in a variety of public locations is acceptable [17, 18]. Acker [6] and Li et al. [15] found that American men’s and women’s reactions and attitudes toward public breastfeeding did not differ significantly. Given these mixed results, we included gender as a variable of interest in the present study, but did not make an a priori hypothesis regarding its role in reactions to public breastfeeding. Specifically, we were interested in whether participants’ gender would have an effect on their evaluations of women in general (RQ2a), of breastfeeding (vs. non-breastfeeding) women (R2Qb), and of breastfeeding women in public (vs. private) locations (RQ2c).

#### Sexual comfort

Although breastfeeding is not a sexual act, some people may perceive it as such, especially because female breasts are often sexualized. Thus, one’s comfort level with sexual topics and reactions to sexual stimuli may influence their evaluations of breastfeeding (public breastfeeding in particular). This comfort level has been conceptualized as a dimension ranging from erotophobia (negative affective reactions, avoidance of sexual topics) to erotophilia (positive affective reactions, attraction to sexual topics [28, 29]). Very little research has examined the association between erotophobia and reactions to breastfeeding. Forbes et al. [10] found that both American women and men who scored high on erotophobia tended to have more negative attitudes toward breastfeeding mothers compared to bottle-feeding mothers; however, Forbes and colleagues did not examine the location of breastfeeding as a mitigating factor. Therefore, we predicted that, compared to people with higher levels of sexual comfort (i.e., erotophilic attitudes), people with lower levels of sexual comfort (i.e., erotophobic attitudes) would evaluate breastfeeding women less favorably than non-breastfeeding women (H3a), and this would be especially evident when the breastfeeding takes place in a public (vs. private) location (H3b).

#### Sexist attitudes

Because breastfeeding is an activity that only women engage in, generalized negative attitudes toward women (i.e., sexism) may be related to attitudes toward (public) breastfeeding. This may be particularly true for *benevolent* sexism, which reflects idealization of the traditional female gender role [30]. Forbes and colleagues [10] found that men who scored high on benevolent sexism tended to evaluate breastfeeding mothers more favorably than bottle-feeding mothers on several dimensions (as breastfeeding exemplifies the female gender role). However, Acker [6] found that benevolent sexism among male participants was moderated by location; men who scored high on benevolent sexism tended to express approval for private breastfeeding but disapproval of public breastfeeding (perhaps because public breastfeeding violates the expectation of female modesty). In the current study, we hypothesized that people who endorse benevolent sexism to a higher degree would evaluate breastfeeding more favorably than people who endorse benevolent sexism to a lesser degree (H4a); however, they would evaluate public breastfeeding less favorably than private breastfeeding (H4b).

The role of *hostile* sexism (the general dislike and distrust of women [30]) in attitudes toward breastfeeding is less clear. Forbes et al. [10] found positive associations among men between hostile sexism and evaluations of breastfeeding mothers compared to bottle-feeding mothers (but less so than benevolent sexism). In contrast, Acker [6] found that participants scoring high on hostile sexism tended to rate breastfeeding mothers less favorably than those low on hostile sexism, but this did not interact with location (unlike benevolent sexism). In both studies, neither hostile nor benevolent sexism played a significant role in women’s evaluations of breastfeeding. Because of these mixed findings, we did not make a priori hypotheses about the role of hostile sexism in evaluations of breastfeeding women; however, we examined whether hostile sexism would have an effect on evaluations of breastfeeding women in general (RQ4c) and of breastfeeding women in public (vs. private) locations (RQ4d).

#### Breastfeeding knowledge

Knowledge of breastfeeding is likely to influence people’s evaluations of public breastfeeding. Generally speaking, most people do not encounter women breastfeeding in public on a regular basis, nor do they see such behavior depicted in media frequently. Furthermore, many people (perhaps especially those without children) may not be well informed of the benefits of breastfeeding and the frequency with which infants need to eat. A lack of this knowledge may contribute to negative reactions to public breastfeeding. This is supported by Magnusson and colleagues [22] who found that greater knowledge of breastfeeding was correlated with positive perceptions of breastfeeding images (both private and public breastfeeding). Furthermore, viewing a television depiction of public (vs. private) breastfeeding produced more support for public breastfeeding [31], suggesting that more media depictions of public breastfeeding may shape attitudes in a positive direction. Therefore, we predicted a two-way interaction between breastfeeding and breastfeeding knowledge, such that people with less breastfeeding knowledge would evaluate breastfeeding women less favorably than would people with more breastfeeding knowledge (H5a), and this would be especially evident for publicly breastfeeding women (compared to privately breastfeeding women; H5b).

#### Breastfeeding experience

Acker [6] examined age and parental status as proxies for breastfeeding experience and reported that American parents tended to rate breastfeeding more favorably than non-parents (this effect did not interact with location). Older participants had more favorable attitudes toward breastfeeding than younger participants did, but this was moderated by location: there was no difference in feelings toward the privately breastfeeding woman, but older participants’ feelings toward the publicly breastfeeding woman were less negative than were younger participants’ feelings. In the U.K., relatively high levels of support for public breastfeeding were found among parents in general, as well as among parents whose children were breastfed and whose children were breastfed in public [27]. Taken together, these results suggest that greater experience with breastfeeding positively influences perceptions of (public) breastfeeding. Thus, we hypothesized that experience with breastfeeding (as defined as having had a child who was breastfed) would be associated with more favorable reactions to breastfeeding (H6a) and in particular public breastfeeding (H6b).

### Current research overview

More experimental examinations of evaluations of public breastfeeding are needed; thus, one goal of the current research was to conceptually replicate Acker’s [6] study to investigate whether attitudes toward public breastfeeding have changed in the years since this work was published. In addition, the current study addressed some of the limitations of Acker’s study by utilizing pictures of two breastfeeding women in two public and two private locations (Acker’s study used one breastfeeding woman in one public and one private location). We operationalized “private” places as those in which a breastfeeding mother could reasonably expect to be out of view from others (e.g., at home or in a restroom stall); conversely, “public” places were operationalized as those in which one could easily be in view of others (e.g., coffee shop, outdoor park bench), regardless of whether other people were actually present. Three of the four places utilized in the current study (park bench, coffee shop, outdoor bench) have been examined in other breastfeeding studies (e.g., [6, 22]). The fourth location (restroom stall) has not been examined in previous literature; however, we included it in the present study because it is a location that breastfeeding women may turn to when they seek privacy while out in public (or they may be asked by others to move to a restroom when breastfeeding in public).

To further expand on Acker’s study, we measured participants’ actual prior breastfeeding experience (Acker used parental status as a proxy for familiarity/experience with breastfeeding), and we included a control condition in which the same women were pictured (in the same locations) but were not breastfeeding. Because exposure of a breast may underlie negative reactions to public breastfeeding [23], and because people are more supportive of public breastfeeding when the woman uses a cover [31], we also manipulated the use of a breastfeeding cover in the current study. Finally, we aimed at examining multiple facets of people’s evaluations. Specifically, we took the approach of dividing people’s evaluations into three categories of responses: their emotional reactions to the women in the photographs (positive and negative), their perceptions of the women, and their behavioral intentions toward the women (which could be indicative of their actual behavior [32]).

To summarize, we formed research questions and hypotheses that fall into six main areas:

#### Group 1 (G1): primary hypotheses and research questions regarding public breastfeeding

- RQ1a: Will people’s evaluations of the women be influenced by whether or not they are breastfeeding?
- H1b: Two-way interaction between location and breastfeeding such that public breastfeeding will be evaluated less favorably than private breastfeeding, but there will be no influence of location observed for non-breastfeeding women.
- H1c: Two-way interaction between cover and breastfeeding such that evaluations of the breastfeeding women will be more favorable when they are covered vs. uncovered. No difference due to the use of a cover is expected for the non-breastfeeding women.
- H1d: Three-way interaction between breastfeeding, cover, and location such that the uncovered publicly breastfeeding women will be evaluated least favorably.

#### Group 2 (G2): research questions regarding possible gender differences

- RQ2a: Will participant gender affect evaluations of the women in general?
- RQ2b: Will participant gender differentially affect evaluations of breastfeeding vs. non-breastfeeding women?
- RQ2c: Will participant gender differentially affect evaluations of publicly vs. privately breastfeeding women?

#### Group 3 (G3): hypotheses regarding sexual comfort level

- H3a: Two-way interaction between sexual comfort level and breastfeeding, such that people who are more (vs. less) comfortable with sexual topics will evaluate breastfeeding women more favorably.
- H3b: Three-way interaction between sexual comfort level, breastfeeding, and location, such that the above two-way interaction will be more pronounced in the public condition than the private condition.

#### Group 4 (G4): hypotheses and research questions regarding sexism

- H4a: Two-way interaction between benevolent sexism and breastfeeding such that people who endorse benevolent sexism to higher (vs. lower) degrees will have more favorable evaluations of breastfeeding women.
- H4b: Three-way interaction between benevolent sexism, breastfeeding, and location such that people who endorse benevolent sexism to higher (vs. lower) degrees will evaluate women more favorably when they are breastfeeding (vs. no breastfeeding) in private (vs. public).
- RQ4c: Will hostile sexism differentially affect evaluations of breastfeeding women compared to non-breastfeeding women?
- RQ4d: Will hostile sexism differentially affect evaluations of breastfeeding women in public vs. private locations?

#### Group 5 (G5): hypotheses regarding knowledge of breastfeeding

- H5a: Two-way interaction between knowledge of breastfeeding and breastfeeding such that those who are more knowledgeable about breastfeeding will evaluate the breastfeeding women more favorably than those who are less knowledgeable about breastfeeding. No differences due to knowledge of breastfeeding are predicted for non-breastfeeding women.
- H5b: Three-way interaction between knowledge of breastfeeding, breastfeeding, and location such that the above two-way interaction will be more pronounced for public breastfeeding than private breastfeeding.

#### Group 6 (G6): hypotheses regarding prior breastfeeding experience

- H6a: Two-way interaction between previous experience with breastfeeding and breastfeeding such that those who have more experience with breastfeeding will evaluate breastfeeding women more favorably compared to those who have less experience with breastfeeding. No differences due to experience with breastfeeding are expected for non-breastfeeding women.
- H6b: Three-way interaction between previous experience with breastfeeding, breastfeeding, and location such that the above two-way interaction will be more pronounced for public breastfeeding than private breastfeeding.

## Methods

### Participants

An a priori power analysis using G*Power 3.1.9.7 [33] indicated that in order to detect a small effect size with 80% power, 550 participants will be needed. In August 2018, a total of 862 participants were recruited from Amazon Mechanical Turk, a crowdsourcing website where participants can complete on-demand tasks and participate in various surveys and studies. The MTurk population has been reported to be fairly representative of the USA general population [34], especially in terms of gender and race [35]. Only participants over the age of 18 and from the USA were recruited as to avoid cultural influences. Participants were compensated 50 cents for their participation.

### Procedure and measures

In the informed consent document, participants were told that the purpose of the study was to investigate how dress and behavior influence perceptions of people. They were informed that they would view photos of people dressed in various ways and engaging in various behaviors and that they would be asked to answer questions about their perceptions of the people in the photos. After giving consent to participate, participants were randomly assigned to view a single picture of a woman. The woman in the photo was either breastfeeding or on her phone; she was either not covered or covered with a gray scarf; and she was either in a private location (living room or bathroom stall) or public location (Starbucks or park). For stimulus sampling purposes, two different women were used as models in the photos; thus, participants were assigned to view one of 16 total photos (see Additional file 1 for sample photos used in the study).

After viewing the photo, participants were asked to rate their emotional reactions toward the woman, their perceptions of the woman, and their behavioral intentions to interact with the woman. They were then asked to complete a series of scales assessing their sexist attitudes, their sexual comfort, their knowledge about breastfeeding, their experience with breastfeeding, and a measure of desirable responding. Participants then answered demographic questions. Following completion of the experiment, participants were thanked and debriefed to clarify the true purpose of the study.

#### Emotions

Participants’ positive and negative emotions associated with the depicted woman were measured with the feelings scale [36], as adapted by Forbes et al. [10]. A principal component factor analysis of the 13 items, using varimax rotation, was performed to confirm the two subcategories of the scale and accounted for 57.15% of the variance. The emotions “bored” and “comfortable” loaded weakly, so they were removed from the scales, resulting in a 4-item positive emotion scale and 7-item negative emotions scale. Sample negative emotions include “disgusted,” “angry,” and “nauseated,” whereas sample positive emotions include “excited” and “entertained.” The emotions were rated on a 6-point Likert scale ranging from 1 = *not at all* to 6 = *very*. An average score was calculated for each participant (negative emotions α = .90; positive emotions α = .75).

#### Perceptions

Participants’ perceptions of the depicted woman were measured with the perceptions scale (adapted from Marks and Fraley’s work [37]), which consists of 20 items rated on a 1 = *strongly disagree* to 5 = *strongly agree*. Sample items include “This person is careless” (reversed coded), “This person has a bright future,” and “This person is good at analyzing situations.” An average score was calculated for each participant (α = .95).

#### Behavioral intentions

Participants’ behavioral intentions to interact with the depicted woman were measured with the behavioral intentions scale [38]. Sample items include “I want to spend time with this person” and “I want this person as a neighbor.” The behavioral intentions scale consists of 10 items rated on a 1 = *not at all* to 7 = *very much*. An average score was calculated for each participant (α = .95).

#### Sexual comfort

Participants’ comfort level with sexual topics was assessed using the 20-item Sexual Opinion Survey [29]. Participants rated their agreement on a 7-point scale ranging from 1 = *strongly disagree* to 7 = *strongly agree*. Sample items include “Masturbation can be an exciting experience,” “The thought of engaging in unusual sexual practices is highly arousing,” and “Almost all pornographic material is nauseating” (reversed coded). An average score was calculated for each participant (α = .92).

#### Sexist attitudes

Participants’ sexist attitudes were assessed using the 22 item Ambivalent Sexism Inventory [30]. Participants rated their agreement with statements concerning men and women and their relationships in contemporary society on a 6-point scale ranging from 1 = *strongly disagree* to 6 = *strongly agree*. The inventory consists of two subscales: the hostile sexism scale, which includes items such as “Women are too easily offended” and “Women seek to gain power by getting control over men,” and the benevolent sexism scale, which includes items such as “Women should be cherished and protected by men” and “No matter how accomplished he is, a man is not truly complete as a person unless he has the love of a woman.” An average score was calculated for each participant (hostile sexism α = .92; benevolent sexism α = .88).

#### Breastfeeding knowledge

Participants’ breastfeeding knowledge was assessed using selected items from the 17-item Iowa Infant Feeding Attitude Scale [39]. We selected the 10-items from the scale that assessed breastfeeding knowledge related to the cost of infant feeding and nutrition. Participants rated their agreement regarding various statements relating to breastfeeding on a 5-point scale ranging from 1 = *strong disagreement* to 5 = *strong agreement*. Sample items include “Breast milk is less expensive than formula” and “Breast milk is the ideal food for babies.” An average score was calculated for each participant (α = .72).

#### Previous breastfeeding experience

Participants’ personal experience with breastfeeding was also assessed. Specifically, they were asked, “If you are a parent, was at least one of your children breastfed?” Responses were coded as 0 = *no* and 1 = *yes (*2 = *I am not a parent)*.

#### Social desirability

In order to account for participants’ social desirability, the 13-item Marlowe-Crowne Scale [40] was administered. Due to a mistake, only 12 items were included in the survey. Sample items include, “I sometimes feel resentful when I don’t get my way” and “I am always courteous, even to people who are disagreeable.” An average score was calculated for each participant (α = .75).

## Results

Of the original sample of 862, 182 participants were removed for not completing the survey in full, 12 participants were removed because they completed the survey more than once (their second attempt was removed), and 162 participants were removed for failing to correctly answer one or more manipulation check questions (i.e., Was the woman depicted in the photo breastfeeding? Where was the woman in the photo depicted? Was the woman in the photo wearing a cover/scarf?). The final sample consisted of 506 participants (63.1% women) with a median age of 34 years old (*M* = 36.86, *SD* = 12.25). The sample was predominantly heterosexual (86.8%), Caucasian (77.7%), Christian/Catholic (53%), and liberal/very liberal (43.8%). The majority of the sample identified as parents (56.1%). See Table 1 for full frequency statistics of the sample’s demographics.

**Table 1:** Frequency Statistics of the Sample’s Demographic Characteristics

Category	Frequency	Percent
	*n*	*%*
Gender Identity		
Male	196	38.7
Female	310	61.3
Age		
18–20	9	1.8
21–30	182	36.0
31–40	160	31.6
41–50	73	14.4
51–60	45	8.9
61–70	32	6.2
71–73	4	0.8
Sexual Orientation		
Heterosexual	439	86.8
Homosexual	12	2.4
Bisexual	42	8.3
Other	13	2.6
Ethnicity		
White / Caucasian	393	77.7
Hispanic / Latino	26	5.1
Black / African American	35	6.9
Asian / Asian American	37	7.3
Native American / American Indian	2	0.4
Mixed	12	2.4
Other	1	0.2
Political Views		
Very Liberal	67	13.2
Liberal	155	30.6
Moderate	123	24.3
Conservative	107	21.1
Very Conservative	40	7.9
No Opinion	13	2.6
Religious Views		
Agnostic	95	18.8
Atheist	78	15.4
Catholic	87	17.2
Christian	181	35.8
Hindu	1	0.2
Jewish	14	2.8
Muslim	1	0.2
Other	48	9.5
Parental Status		
Parent	284	56.1
Not parent	222	43.9

*Note*. There is one missing data point for the age, political and religious variables

An independent t-test revealed no significant differences between the two women that were depicted in the photos (positive emotions, *t* = −.18, *p* = .86; negative emotions, *t* = − 1.92, *p* = .06; perceptions, *t* = −.32, *p* = .75; behavioral intentions, *t* = −.75, *p* = .46). Therefore, the mother variable was collapsed across the variables of interest (breastfeeding, location, and cover). To begin our investigation, a correlational analysis was performed, once for the whole sample and once for the subsample of parents (see Tables 2 and 3, for correlations and descriptive statistics for key variables). To examine the hypothesized effects of G1-G6, a set of hierarchical regressions was conducted. Each of the hierarchical regressions was performed four times, once for each of the dependent variables: positive emotions toward the woman, negative emotions toward the woman, perceptions of the woman, and behavioral intentions toward the woman. To control for a possible type one error, we decided to use a more conservative alpha level of 0.01 to indicate significance. The categorical conditions were dummy coded such that “female participant,” “public location,” “breastfeeding,” and “covered” conditions were coded 1, and “male participant,” “private location,” “not breastfeeding,” and “not covered” conditions were coded 0. Centered versions (based on scale means) of hostile sexism, benevolent sexism, sexual comfort, breastfeeding knowledge, and social desirability scales were created. There were no multicollinearity violations with any of the reported below results; all variance inflation factors were under 10 [41].

**Table 2:** Descriptive statistics and correlations between variables for men (above the diagonal) and women (below the diagonal)

	Sexual Comfort	Hostile Sexism	Benevolent Sexism	Breastfeeding Knowledge	Positive Feelings	Negative Feelings	Perceptions	Behavioral Intentions
Sexual Comfort	–	−0.24**	− 0.22**	0.21**	− 0.01	− 0.32***	0.16*	0.08
Hostile Sexism	−0.28***	–	0.34***	−0.19**	0.25***	0.31***	− 0.21**	− 0.14
Benevolent Sexism	− 0.33***	0.50***	–	− 0.08	0.28***	0.20**	0.10	0.13
Breastfeeding Knowledge	−0.02	− 0.09	0.07	–	− 0.28***	−0.29***	0.02	−0.07
Positive Feelings	−0.02	0.15*	0.18**	−0.08	–	0.57***	0.16*	0.35***
Negative Feelings	−0.12	0.21***	0.15*	−0.17**	0.31***	–	−0.18*	0.07
Perceptions	0.09	−0.14*	−0.02	0.19***	0.23***	−0.37***	–	0.80***
Behavioral Intentions	−0.001	−0.10	0.02	0.14*	0.20***	−0.27***	0.81***	–
Mean	4.55	2.99	3.21	3.58	1.67	1.43	3.36	3.99
SD	1.17	1.16	1.03	0.56	0.86	0.79	0.61	1.49
Range	1.00–7.00	1.00–6.00	1.00–5.82	1.90–5.00	1.00–5.00	1.00–5.00	1.00–5.00	1.00–7.00
Scale	1–7	1–6	1–6	1–5	1–6	1–6	1–5	1–7

*Note*. Descriptive statistics are for the full sample. Gender coded: male = 0, female = 1

*p ≤ .05, **p ≤ .01, ***p ≤ .001

Because of missing data on individual items, *n*s for the male sub-sample range from 170 to 196, and *n*s for the female sample range from 262 to 308

**Table 3:** Descriptive statistics and correlations between variables for participants who identified as male parents (above the diagonal) and female parents (below the diagonal)

	Sexual Comfort	Hostile Sexism	Benevolent Sexism	Breastfeeding Knowledge	Positive Feelings	Negative Feelings	Perceptions	Behavioral Intentions	Breastfed Child
Sexual Comfort	–	−0.26*	−0.26*	0.19	−0.08	− 0.31**	0.27**	0.14	0.11
Hostile Sexism	−0.24**	–	0.45***	−0.30**	0.43***	0.32**	−0.12	0.03	−0.08
Benevolent Sexism	−0.27***	0.45***	–	−0.14	0.29**	0.20	0.04	0.05	−0.12
Breastfeeding Knowledge	0.06	−0.10	0.03	–	−0.48***	− 0.40***	0.08	− 0.16	0.27**
Positive Feelings	−0.03	0.17*	0.20*	−0.15	–	0.66***	0.08	0.34***	−0.06
Negative Feelings	−0.06	0.28***	0.24**	−0.25**	0.50***	–	−0.14	0.16	−0.001
Perceptions	0.05	−0.19*	−0.10	0.21**	0.17*	−0.25**	–	0.79***	0.15
Behavioral Intentions	0.01	−0.15*	−0.08	0.16*	0.17*	−0.18*	0.80***	–	0.11
Breastfed Child	−0.05	−0.003	− 0.001	0.42***	− 0.11	−0.06	0.05	0.10	–
Mean	4.35	3.14	3.40	3.62	1.68	1.48	3.38	4.14	–
SD	1.19	1.10	1.00	0.59	0.89	0.86	0.59	1.49	–
Range	1.00–6.70	1.00–5.91	1.00–5.82	1.90–5.00	1.00–5.00	1.00–5.00	1.25–5.00	1.00–7.00	–
Scale	1–7	0–5	0–5	1–5	1–6	1–6	1–5	1–7	

*Note*. Descriptive statistics are for the full sub-sample of parents. Gender coded: male = 0, female = 1

*p ≤ .05, **p ≤ .01, *** *p* ≤ .001

Because of missing data on individual items, *n*s for the male sub-sample range from 82 to 102, and *n*s for the female sample range from 156 to 182

### Group 1: primary hypotheses and research questions regarding public breastfeeding

To examine hypotheses about public breastfeeding in general, breastfeeding, location, and cover variables were entered in Step 1 (along with a centered version of social desirability). The hypothesized two-way interactions were entered in Step 2, and the hypothesized three-way interaction was entered in Step 3.

Given the inconsistent previous research findings regarding the acceptance of breastfeeding in general, we examined whether there was a main effect for breastfeeding (RQ1a). There was a main effect for breastfeeding such that participants had fewer positive emotions (*β =* −.15, *p* < .01, *f*^*2*^ *=* .02; see Cohen’s [42] effect size interpretations), more favorable perceptions (*β =* .32, *p* < .001, *f*^*2*^ *=* .13), and greater behavioral intentions to interact with the breastfeeding woman (*β =* .33, *p* < .001, *f*^*2*^ *=* .13), compared to the non-breastfeeding woman. None of the hypothesized interactions regarding public breastfeeding (H1b, H1c, and H1d) were supported.

### Group 2: research questions regarding possible gender differences

Similar regression analyses were performed again with only participant gender, breastfeeding, and location entered in Step 1. The appropriate two-way interaction and three-way interaction were entered in Step 2 and 3 (respectively). Given the inconsistent previous research findings regarding the gender, we examined whether there was a main effect for participant gender (RQ2a). There was a main effect for participant gender such that female participants had fewer positive emotions (*β =* −.31, *p* < .001, *f*^*2*^ *=* .11), fewer negative emotions (*β =* −.21, *p* < .001, *f*^*2*^ *=* .05), and more favorable perceptions of the woman (*β =* .14, *p* < .01, *f*^*2*^ *=* .02), compared to male participants. None of the other research questions regarding gender and breastfeeding were supported (RQ2b and RQ2c).

### Group 3: hypotheses regarding sexual comfort level

Similar regression analyses were performed again with only breastfeeding, location, and a centered version of sexual comfort entered in Step 1. The appropriate two-way interaction and three-way interaction were entered in Step 2 and 3 (respectively).

**Fig. 1:**
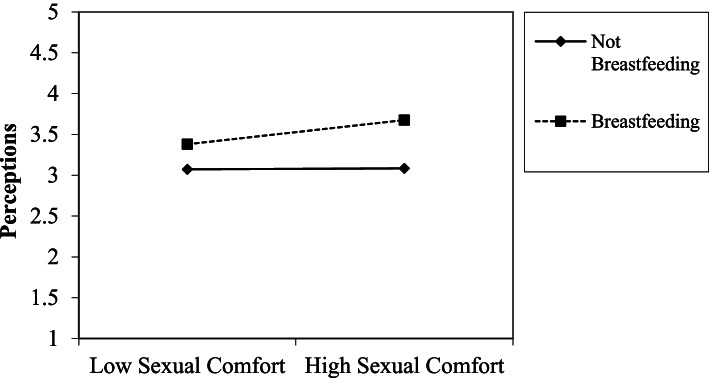
An interaction between participants’ sexual comfort and breastfeeding condition (image of breastfeeding woman vs. not breastfeeding woman) for the perceptions scale

We predicted a two-way interaction between sexual comfort and breastfeeding; specifically, we predicted that individuals who are more comfortable with sexual topics would evaluate breastfeeding women more favorably, compared to individuals who are less comfortable with sexual topics. There was a significant two-way interaction between sexual comfort and breastfeeding for perceptions (*β =* .18, *p* < .01, *f*^*2*^ *=* .02). Specifically, participants who were more sexually comfortable had more favorable perceptions of the breastfeeding woman compared to participants who were less sexually comfortable (*t* = 3.25, *p* = 0.001; see Fig. 1). The hypothesized three-way interaction between sexual comfort, breastfeeding, and location was not supported.

### Group 4: hypotheses and research questions regarding sexism

Similar regression analyses were performed again with only breastfeeding, location, and centered versions of hostile sexism and benevolent sexism attitudes entered in Step 1. The appropriate two-way interactions and three-way interactions were entered in Step 2 and 3 (respectively). Because previous research has found that sexism plays a role in men’s (but not women’s) evaluations of breastfeeding [6, 10], the analyses were performed twice, once for male participants and once for female participants. There were no two-way interactions between benevolent sexism or hostile sexism with breastfeeding (H4a and RQ4c, respectively). There were also no three-way interactions between benevolent sexism or hostile sexism with breastfeeding and location (H4b and RQ4d, respectively).

There was, however, a main effect for benevolent sexism such that female participants who endorsed benevolent sexism to a greater degree had more positive emotions (*β =* .20, *p* < .01, *f*^*2*^ *=* .04) toward the woman in general, compared to those who endorsed benevolent sexism to a lesser degree. Moreover, there was a main effect for hostile sexism such that male participants who endorsed hostile sexism to a greater degree had more negative emotions (*β =* .27, *p* < .01, *f*^*2*^ *=* .11), less favorable perceptions of (*β =* −.26, *p* < .01, *f*^*2*^ *=* .04), and lower desire to want to interact with the woman in general (*β =* −.20, *p* < .01, *f*^*2*^ *=* .02), compared to those who endorsed hostile sexism to a lesser degree.

### Group 5: hypotheses regarding knowledge of breastfeeding

**Fig. 2:**
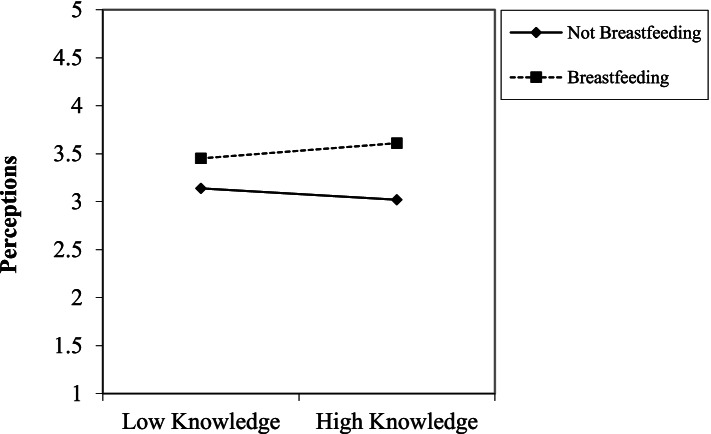
An interaction between participants’ knowledge of breastfeeding and breastfeeding condition (image of breastfeeding woman vs. not breastfeeding woman) for the perceptions scale

Similar regression analyses were performed again with only breastfeeding, location, and a centered version of breastfeeding knowledge entered in Step 1. The appropriate two-way interaction and three-way interaction were entered in Step 2 and 3 (respectively). We predicted a two-way interaction between knowledge of breastfeeding and breastfeeding; specifically, we predicted that individuals who are more knowledgeable about breastfeeding would have more favorable evaluations of breastfeeding women compared to individuals who are less knowledgeable about breastfeeding (H5a). There was a two-way interaction between breastfeeding knowledge and breastfeeding for perceptions (*β =* .18, *p* < .01, *f*^*2*^ *=* .04). Specifically, participants with greater breastfeeding knowledge had more favorable perceptions of the breastfeeding woman compared to participants with less breastfeeding knowledge (*t* = 2.84, *p* = 0.07; see Fig. 2). The hypothesized three-way interaction between breastfeeding knowledge, breastfeeding, and location (H5b) was not supported.

### Group 6: hypotheses regarding prior breastfeeding experience

To examine the role of previous experience with breastfeeding, we analyzed responses from a sub-sample of participants who reported being parents. Parents’ previous experience with breastfeeding was entered as a categorical variable (coded as: 1 = had at least one child who was breastfed; 0 = did not have a child who was breastfed). We predicted a two-way interaction between previous experience with breastfeeding and breastfeeding; specifically, those who have more experience with breastfeeding (being a parent and having had at least one child breastfed) would have more favorable evaluations of breastfeeding women compared to those who have less experience with breastfeeding (parents who haven’t had their child(ren) breastfed (H6a)).

**Fig. 3:**
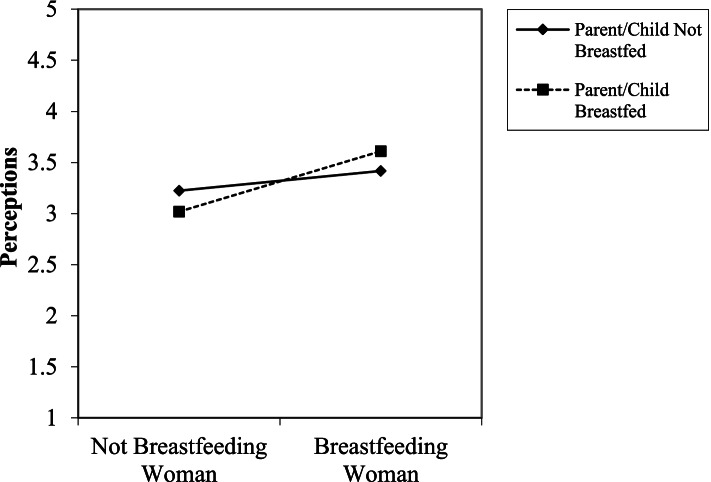
An interaction between parents’ breastfeeding experience (child was breastfed vs. child was not breastfed) and breastfeeding condition (image of breastfeeding woman vs. not breastfeeding woman) for the perceptions scale

There was a two-way interaction between previous breastfeeding experience and breastfeeding for perceptions (*β =* .34, *p* < .01, *f*^*2*^ *=* .15). However, the predicted pattern was not observed. Instead, participants who were parents and had their child(ren) breastfed had more favorable perceptions of the breastfeeding woman than the non-breastfeeding woman (*t* = 5.59, *p* < .001; See Fig. 3). The hypothesized three-way interaction between breastfeeding familiarity, breastfeeding, and location (H6b) was not supported.

## Discussion

The current project aimed to examine evaluations of breastfeeding women by analyzing self-reported responses to an image of a woman breastfeeding (or not) in various locations. Primarily we were interested in whether publicly breastfeeding women would be evaluated differently compared to privately breastfeeding women. Furthermore, we examined three different explanations for the possible differences in people’s evaluations of breastfeeding women: sexualization of the female breast, sexist attitudes, and familiarity with breastfeeding.

### Factors influencing perceptions of breastfeeding

Based on our findings, it appears that familiarity with breastfeeding, defined both as knowledge of breastfeeding and actual firsthand experience (having at least one child who was breastfed), influenced people’s evaluations of breastfeeding. In particular, people who were more knowledgeable of breastfeeding had marginally more favorable perceptions of breastfeeding women compared to people who were less knowledgeable. This is supported by previous research that also found greater knowledge related to favorable perceptions of breastfeeding [22]. In addition, participants with firsthand experience with breastfeeding had more favorable perceptions of the breastfeeding (vs. non-breastfeeding) woman. Our findings expand on Acker’s work [6] which found that parents had more favorable perceptions of breastfeeding compared to non-parents. While Acker relied on the assumption that parents would be more familiar with breastfeeding than non-parents (essentially using parenthood as a proxy for breastfeeding familiarity), we specifically assessed breastfeeding experience among parents in our sample.

A second component that appears to influence people’s evaluations of breastfeeding women was people’s sexual comfort. People who had greater sexual comfort had more favorable perceptions of breastfeeding women than people who had lesser sexual comfort. This is supported by previous research that also found lower sexual comfort was associated with less favorable attitudes toward breastfeeding mothers compared to bottle-feeding mothers [10]. Our findings expand on Forbes and colleagues’ work [10] by examining how sexual comfort specifically affects evaluations of breastfeeding vs. not breastfeeding women.

Although sexual comfort appeared to be related to evaluations of breastfeeding women, the other factors associated with the sexualization of the female breast (the presence of a cover and participants’ gender) did not affect these evaluations. Even though some previous research illustrates that some of the negative bias toward breastfeeding women is similar to the bias against women whose breasts are sexualized [9], our results did not find any evidence for different evaluations of breastfeeding women based on their degree of coverage or location. We did, however, find a main effect for cover for perceptions (*β* = .12, *p* < .01, *f*^*2*^ = .02); whether breastfeeding or not, perceptions of women who were covered by the scarf were more favorable than those of women who were not covered. This effect was not hypothesized, but it may suggest that American adults generally find it more acceptable for women’s chests and breasts to be covered. In regard to gender, we found that compared to male participants, female participants had fewer positive and negative emotions, and more favorable perceptions of women in general. However, participants’ gender did not influence their evaluations of breastfeeding. Given that some previous studies have similarly not detected an effect of gender on breastfeeding perceptions [6, 15] and that others have yielded mixed results [16–18], our findings are not surprising.

Finally, the third explanation of differing evaluations of breastfeeding that focused on sexist attitudes was not supported by the current study. Although, not surprisingly, people with more sexist attitudes evaluated the women differently than those with less sexist attitudes, the patterns did not differ based on whether the woman was breastfeeding or not. Previous research has demonstrated that among men, benevolent sexism influences breastfeeding attitudes. In particular, Acker [6] found that compared to women, men who endorsed benevolent sexism had less favorable perceptions of publicly breastfeeding women. Forbes et al. [10] found that men who endorsed benevolent sexism had more favorable evaluations of breastfeeding women (compared to bottle-feeding women). The lack of interaction between breastfeeding and sexist attitudes in the current study could indicate that sexist attitudes no longer significantly influence people’s evaluations of breastfeeding women. Indeed, a longitudinal examination of sexism revealed decreases in sexism over time (New Zealand sample [43]).

### Breastfeeding location

Additionally, it is important to note that our results did not indicate an effect of breastfeeding location on people’s evaluations of breastfeeding women. This is in contrast to Acker [6] and Magnusson et al. [22], in which evaluations were more favorable in response to private than public breastfeeding images. There are two possible explanations for this discrepancy. It is possible that in the years since Acker’s and Magnusson and colleagues’ studies (Magnusson collected their data in 2013) were conducted, Americans’ acceptance of public breastfeeding has increased significantly, and their evaluations of breastfeeding are no longer impacted by where the breastfeeding occurs. This possible shift in attitudes is perhaps driven, in part, by the increasing use of social media; many mothers, including celebrities who have large social media audiences, have posted pictures of themselves breastfeeding in an effort to normalize the act of breastfeeding in public. Others have taken to social media to speak out against negative reactions they encountered when breastfeeding in public (e.g., being asked to leave a restaurant despite the legal right to breastfeed there). Breastfeeding “sit-ins” have been organized via social media to show support for breastfeeding mothers and protest the mistreatment of breastfeeding women. Although these occurrences provide only anecdotal evidence, they suggest that there has been more attention paid and conversations centered on public breastfeeding in recent years, perhaps contributing to a shift toward more favorable attitudes; however, more longitudinal studies to examine such shifts in breastfeeding attitudes are needed. Additionally, qualitative studies of breastfeeding attitude change may provide further evidence of whether attitudes have shifted in a favorable direction.

Alternatively, it is possible that the way we operationally defined “public” and “private” locations caused some potential noise in our data. Specifically, our definitions of private and public locations were focused on the degree of privacy the woman has (i.e., being alone), and not on whether the general location is a private one. For example, one of the locations we used for the private condition was a bathroom stall. It was clear that the bathroom stall was in a public bathroom, but the woman was alone in the stall, thus making it a private location. However, some participants may have viewed and evaluated this condition as more public than private.

Magnusson and colleagues [22] speculated that the perception of privacy may be more important than the specific location or setting when they (unexpectedly) found that images of public breastfeeding were evaluated less favorably when other people (especially men) were depicted near the breastfeeding woman than when the breastfeeding woman was depicted alone (similar results have been found among U.K. adults [44]). They speculated that unfavorable evaluations of public breastfeeding may stem more out of concern for the discomfort of oneself and others rather than a concern with the breastfeeding woman. Notably, none of the images used in the current study included people other than the breastfeeding woman, which may explain the differences in results between the current study and Magnusson et al. [22]. Future research should manipulate the presence or absence of other people in evaluating public breastfeeding to examine the relative importance of location and privacy. Clarifying the source of potential discomfort or negative reactions to public breastfeeding is important, as it may shed light on ways to promote acceptance of public breastfeeding, among both mothers and non-mothers alike. Ultimately, this may improve breastfeeding rates as mothers’ negative attitudes about breastfeeding in public are linked to earlier cessation of breastfeeding [7].

### The measurement of attitudes and the importance of perceptions

To examine public breastfeeding attitudes as comprehensively as possible, we assessed and examined multiple facets (affective, cognitive, and behavioral) of attitudes. Specifically, we evaluated people’s emotions (positive and negative), perceptions, and behavioral intentions. Examining our findings, it is clear that the cognitive component of people’s attitudes, their perceptions, was the most prominent component in terms of significant differences due to experimental condition and the other factors (gender, sexual comfort, sexism, breastfeeding familiarity). Differences in perceptions were observed in all six of the hypothesis/research question groups (whereas differences in emotions were observed in three of the six groups, and differences in behavioral intentions were observed in two of the six groups). This suggests that perceptions are perhaps a more malleable or influential component of breastfeeding attitudes, and efforts to increase support for public breastfeeding should target this component. It should be noted that behavioral intentions were used as a proxy for behavior, and the current study did not examine actual behavior. Although behavioral intentions and behavior are associated both correlationally and causally [45], future research should examine people’s behavioral responses to women who are publicly breastfeeding (e.g., verbal comments, eye contact, facial expression, etc.) to better understand their attitudes toward public breastfeeding.

### Additional limitations and future directions

Results of the experiment should be considered in light of additional limitations. One possible limitation is related to the feelings scale we used. This scale was adapted from Forbes and colleagues’ [10] study that utilized the Smeaton and Byrne’s Feelings Scale [34] to assess emotions toward breastfeeding vs. bottle-feeding women. Although the scale encompassed both a positive emotions subscale and a negative emotions subscale, in our experiment these two subscales were positively correlated (*r* = .59, *p* < .001). This indicates that interpretation of the valence of the emotions expressed by our participants should be done with caution. Instead of focusing on what type of emotions were felt by the participants, perhaps we should focus on the level of emotionality felt in general. In addition, some of the individual items included in these scales were arguably not the best suited for our purposes. For example, “entertained” and “excited” (positive emotion items) and “afraid” and “depressed” (negative emotion items) are not likely to be common emotional responses to the act of breastfeeding. This feelings scale could have benefited from inclusion of other items that better tap into people’s responses to breastfeeding, perhaps with less intense emotions (e.g., including “happy” in the positive emotion sub-scale).

Additionally, our images and sample could benefit from greater diversity. The two women in the images used in the current study were White and Hispanic. These women were recruited from a region that is predominantly White and Hispanic, so although these women are representative of the community they were recruited from, they do not represent all breastfeeding women. Additionally, our sample was primarily Caucasian (77.7%). Given that there are differences in breastfeeding rates across ethnic and racial groups [46, 47], it would be worthwhile to examine how more racially and ethnically diverse American samples evaluate breastfeeding women of different racial and ethnic backgrounds, particularly African American women as they tend to report the lowest rates of breastfeeding [48]. Similarly, future research should replicate this study in other countries, as we cannot assume that results from this American sample generalize broadly given that breastfeeding attitudes, norms, and practices differ widely across countries [7, 49, 50] and are influenced by social and cultural contexts.

### Practice implications

A woman’s comfort level with breastfeeding in public has been shown to influence her intention to exclusively breastfeed [3] as well as the continuation or discontinuation of breastfeeding [8], and it seems likely that women will feel more comfortable breastfeeding in public when they perceive that others support that behavior (i.e., when they feel they can breastfeed in public without being met with negative reactions from others). Thus, it would stand to reason that increasing public support for breastfeeding in general would be beneficial. These efforts could include better education of the general public about the health benefits of breastfeeding and the feeding patterns of infants.

Additionally, support for public breastfeeding may increase if people become more familiar with public breastfeeding through an increase in breastfeeding images and storylines in news media, social media, and entertainment media. Consistent with this suggestion, Foss and Blake [31] found that support for public breastfeeding was higher among people who had recently watched a TV clip depicting breastfeeding in a public (vs. private) location. In addition, Newell and colleagues [44] reported a small increase in the favorability of public breastfeeding attitudes after participants briefly viewed images of public breastfeeding, suggesting that these attitudes can be shaped by mere exposure. Findings such as this are an important reminder that breastfeeding attitudes are malleable, and the discomfort that some people may experience when seeing breastfeeding in public may be diminished if this behavior was more familiar to them (i.e., if they saw it more often). As others have noted, increasing the visibility of breastfeeding in public and in the presence of others may shift societal attitudes in a favorable direction that ultimately supports women’s continuation of breastfeeding [24, 44, 51].

Importantly, the results of the current study could provide reassurance to breastfeeding women that they are supported, at least tacitly, by others. Specifically, the breastfeeding woman was evaluated more positively than the non-breastfeeding woman, and this did not differ depending on location (private vs. public). Although stories of negative treatment of breastfeeding women have circulated on social media, these occurrences are not necessarily common and do not reflect the experiences of all breastfeeding women. In fact, in an observational field study of women’s experiences breastfeeding and bottle-feeding in public, an increase in attention from others was noted among women who were breastfeeding, but negative reactions (looks or comments) were very rare [5]. Educating breastfeeding women about these findings may help alleviate their concerns, increase their perceptions of support, and empower them to breastfeed their children wherever they like.

## Conclusion

Our study aimed to examine people’s evaluations of breastfeeding women in public locations. It appears that there are more favorable evaluations of breastfeeding women, especially by those who have more sexual comfort, are more knowledgeable about breastfeeding and those who are parents and had one of their children breastfed. We did not observe any effect of location on such evaluations. It might be that, in general, people’s evaluations of breastfeeding are favorable to the degree that the location of the breastfeeding is not particularly relevant to those evaluations. Future research should replicate these results and attempt to directly observe people’s actual behavior toward publicly breastfeeding women.

## Supplementary Information

**Additional file 1.**

## Data Availability

The dataset used and analyzed during the current study are available from the corresponding author upon reasonable request.
