# Supplementary material for: It’s just a breast: an examination of the effects of sexualization, sexism, and breastfeeding familiarity on evaluations of public breastfeeding
Source: BMC Pregnancy Childbirth. 2022 Feb 12;22:122. doi: 10.1186/s12884-022-04436-1 (PMC8840320; doi:10.1186/s12884-022-04436-1)
Supplement: Supplementary file 1 — Additional file 1. [file 12884_2022_4436_MOESM1_ESM.docx]

Appendix

| 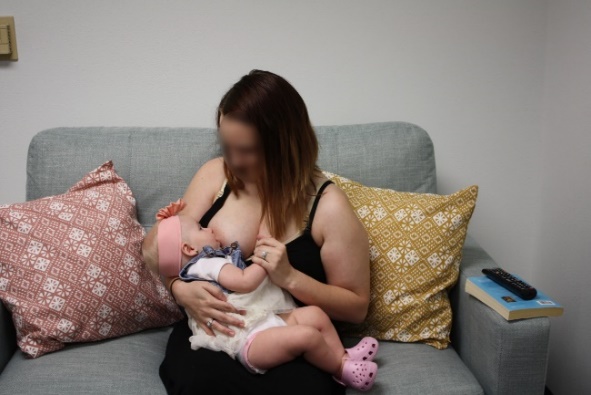  Breastfeeding, No Cover, Living Room | 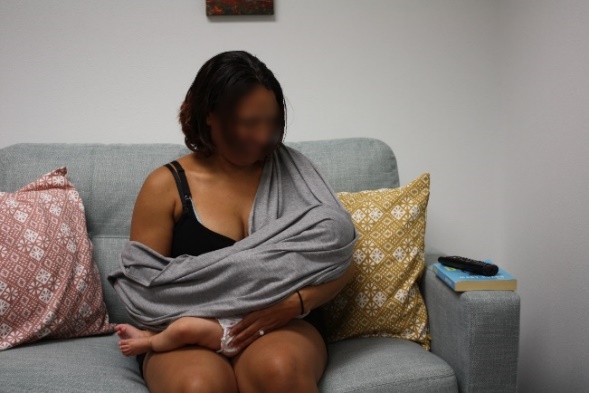  Breastfeeding, Cover, Living Room |
| --- | --- |
| 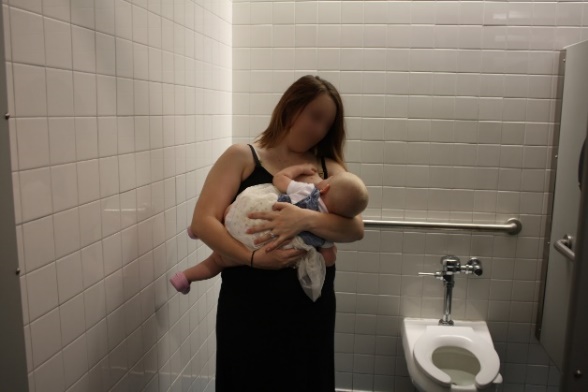  Breastfeeding, No Cover, Restroom Stall | 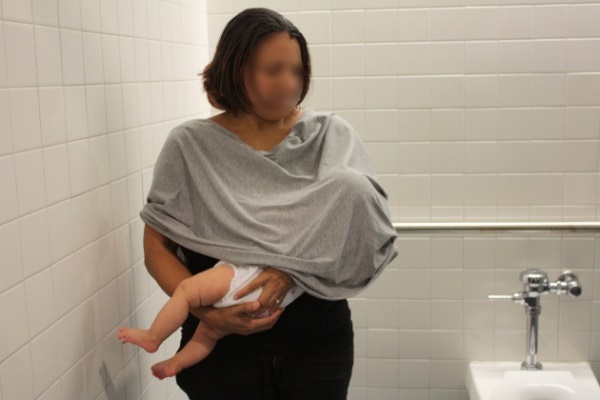  Breastfeeding, Cover, Restroom Stall |
| 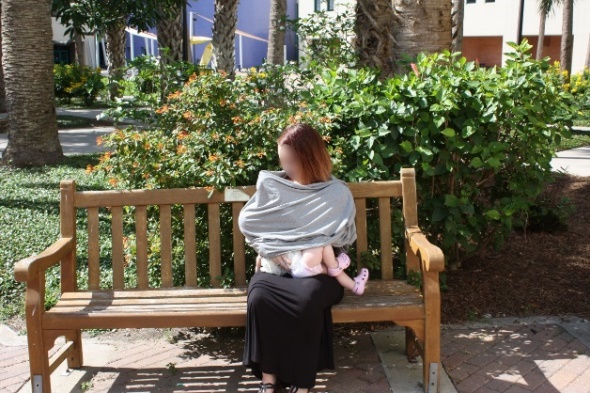  Breastfeeding, Cover, Park | 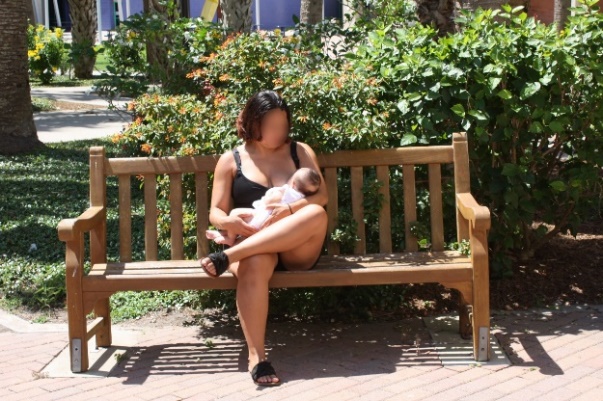  Breastfeeding, No Cover, Park |
| 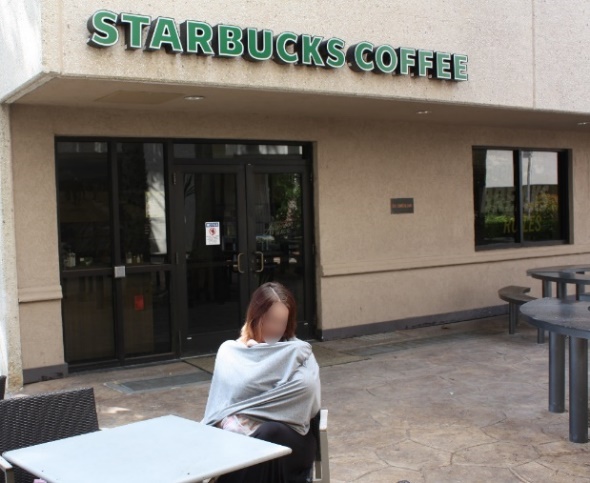  Breastfeeding, Cover, Starbucks | 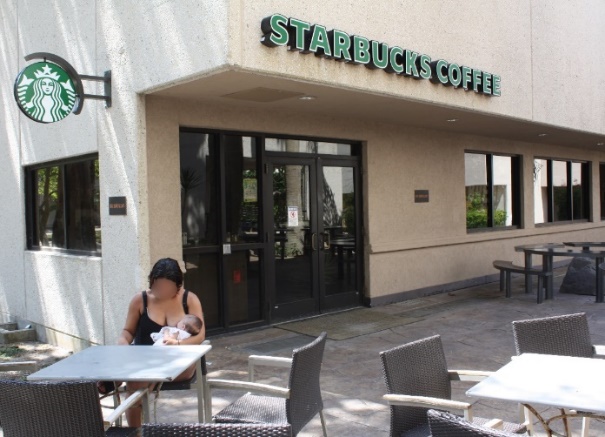  Breastfeeding, No Cover, Starbucks |
